# A Nineteen-Year Cohort Study on the Relationship of Electrocardiographic Findings to All Cause Mortality Among Subjects in The National Survey on Circulatory Disorders, NIPPON DATA80.

**DOI:** 10.2188/jea.15.125

**Published:** 2005-08-30

**Authors:** Hiroshi Horibe, Fumiyoshi Kasagi, Mieko Kagaya, Yasuko Matsutani, Akira Okayama, Hirotsugu Ueshima

**Affiliations:** 1 Aichi Medical University.; 2 Radiation Effects Research Foundation, Hiroshima.; 3 Sugiyama Jogakuen University.; 4 National Cardiovascular Center.; 5 Shiga University of Medical Science.

**Keywords:** Electrocardiography, Minnesota codes, Epidemiology, Cohort studies, Mortality

## Abstract

BACKGROUND: Electrocardiogram (ECG) is one of the most popular tools for daily clinics and health checkup, and has been used for the National Survey on Circulatory Disorders to assess the health status in Japanese people. The meaningfulness to predict mortality from all causes among people with ECG abnormality is to be clarified using national samples.

METHODS: ECG findings recorded among 9,638 subjects for National Survey on Circulatory Disorders 1980 in Japan, were classified using the Minnesota Codes (mc). Their relationships to all cause mortality over 19 years were examined using Cox proportional hazard models adjusting for sex, age, systolic blood pressure, blood glucose, and smoking habits.

RESULTS: Subjects with abnormal Q-QS findings showed significantly high hazard ratios ( 3.71(mc1-1) and 1.57 (mc1-3)) for mortality to the subjects who were free from any major ECG findings. Hazard ratios were 1.37 (mc2-1) to 4.16 (mc2-5) for axis deviation, and 1.34 (mc3-1) to 1.35 (mc3-3) for left high R waves. Those were 1.63 (mc4-3) to 2.59 (mc4-1) for ST depression, and 1.54 (mc5-3) to 2.33 (mc5-1) for T abnormality. The lower the second number of the Minnesota Codes was, the higher hazard ratio was observed in the Q-QS, ST, T codes. The hazard ratios of junction-type ST depression (mc4-4), and low T waves (mc5-4, 5-5) were not significant.

CONCLUSIONS: ECG findings defined by the Minnesota Codes were useful to predict the risk for mortality from all causes even after adjusting for the other major risk factors, and the results supported a usefulness of the ECG for health check-ups.

The major causes of death have been neoplasm, cerebrovascular disease, and ischemic heart disease in these years in Japan. The electrocadiographic (ECG) examination is one of the most efficient and non-invasive methods used to check a condition of the heart. In order to decrease those deaths, Japanese government had worked hard to implement a series of multiphasic health check-ups throughout the country, including ECG examination.^1^ The relevant medical staff and the participants must be interested in the implications of a specific ECG finding for the participant’s prognosis.

For an epidemiologic purpose, the Minnesota Code system was developed by Henry Blackburn and others in 1960,^2^ and revised by Ronald Prineas and others in 1982.^3^ Recently the revised code system was used worldwide to confirm the objectivity of ECG findings.

The Japanese Government performed a series of National Surveys on the Circulatory Disorders almost every ten years.^1^ A research group had been organized to follow-up those participants to clarify the relation of risk factors, such as obesity,^4^ smoking,^5^ serum uric acid,^6^ total cholesterol,^7^ blood pressure,^8^ resting heart rate,^9^ and others.^10^^,^^11^ These participants were from a national stratified random sample throughout the country, not merely nationwide pooled samples by relevant researchers. It was an epoch making achievement to follow these sampled participants and to evaluate their ECG findings objectively with the by Minnesota Code system. The attempt could be achieved by support of Ministry of Health and Welfare (presently, new Ministry of Health, Labour, and Welfare) and extensive cooperation of nearly 300 local public health centers along with collaborators listed in Appendixes I and II. Similar type national samples were available in the United States for National Health and Nutrition Surveys 1971-75, 1976-80, and 1988-94, and many valuable reports presenting year-by-year,^12^^-^^17^ but not yet for extensive ECG findings.

The purpose of this study was to investigate the relationship of ECG findings coded by the Minnesota Code system to deaths of all causes by following-up 19 years of national samples participated in the National Survey on Circulatory Disorders conducted in 1980.

## METHODS

The Ministry of Health and Welfare of the Japanese government carried out the National Survey on Circulatory Disorders in order to clarify the recent status of Japanese people by examining all residents living in the 300 stratified random sample areas selected throughout the country in 1980. A Working Committee charged local public health centers on health check-up in the assigned areas under the guidance for the National Survey (the details described in an Official Report by the Ministry^1^). In 1980, 10,546 people participated in the Survey (Response rate was 79.1%), and the Cohort Study Group on Activity in Daily Living and Prevention of Degradation in Quality of Life among the Elderly followed them up to 1999.

The Cohort Study Group identified 9,638 (4,103 males and 5,535 females) alive or dead people with ECG record successfully by 15 September, 1999. Among them, 118 were lost after the date of moving, but they were definitely alive till the time of moving out according to their official records, “residence registration record” kept in any one of local administrative offices throughout the country. The number of deaths was 1,327 (13.8%), in which 711 deaths were males (16.8%) and 616 deaths were females (11.4%). The detail causes of deaths among this cohort appeared in the previous papers^6^^-^^8^ and a series of annual research reports.^11^ The frequency distribution of deaths did not differ from the national figures in 1980-1990. A Working Group of ECG Coding for the National Survey on Circulatory Disorders carefully coded their ECGs twice by independent coders who belonged to the different universities or institutions in 1980 (Appendix II). If there were any discrepancy in their codes, the third well-experienced coder (one of the authors, HH) made final decision strictly applying the original coding rule (Minnesota Codes 1982).^3^

Major ECG findings were abnormal Q-QS (mc1-1 to 1-3), axis deviation (mc2-1 to 2-5), high R (mc3-1 to 3-4), ST depression (mc4-1 to 4-4), T abnormality (mc5-1 to 5-5), atrio-ventricular conduction defect (mc6-1 to 6-8), intra-ventricular conduction defect (mc7-1 to 7-8), arrhythmia (mc8-1 to 8-9-1), and miscellaneous findings (mc9-1 to 9-3, 9-5). The code 5-5 was an additional Japanese finding for minor T abnormality (T amplitude less than one tenth but over one twentieth of the R amplitude).^18^ The code 8-9-1 was assigned to ectopic beats less than 10 % of recorded beats defined for the Survey. A defined control group was free not only from the relevant code to be analyzed, but also free from any major ECG codes. An abnormal ECG group consisted of subjects with any of major ECG codes, and it was used to analyze the over-all abnormal ECG findings.

In order to apply a multivariate analysis, the Cox proportional hazard model,^19^ we considered the major confounding factors, which were sex, age as of 1980, body mass index, systolic blood pressure, total serum cholesterol, blood glucose, and smoking and drinking habits. However, the hazard ratios of body mass index, drinking habits, and total serum cholesterol did not show statistical significance by a series of pretests and were excluded from further analyses. The number of cigarettes divided smoking habits into 4 levels such as 3 for over 40 cigarettes/day, 2 for 21 to 40, 1 for 1 to 20 cigarettes/day, and 0 for non-smoker, the last of which included ex-smokers.

In order to clarify the effect of deaths close to the date of examination, the Cox hazard ratios for mortality were calculated for deaths in 1980-1999, in 1983-1999, and in1985-1999. A statistical package, Dr’ SPSS^®^ II (a subset of the SPSS sets) was used for this analysis.^20^ The hazard ratios described in the result section were all statistically significant (p<0.05), besides otherwise specified.

## RESULTS

The number of subjects with ECG findings and deaths of all causes are showed in Table 1, along with the death percentages in 19 years. The number of subjects in the reference group was 5,535 and the deaths were 836 (15.1%) during the follow-up period. The number of subjects with any major ECG code was 4,103, and the number of deaths was 1,174 deaths (28.6%).

**Table 1.** Number of subjects and deaths (in parenthesis) among them with electrocardiographic findings by Minnesota code, 19-year follow up, 1980-1999, NIPPON DATA 80 (the death percentages in ***boldface italic*** type)

ECG code	ECG code									Total

	-1	-2	-3	-4	-5	-6	-7	-8	-9	
1 Q-QS code	16 ( 11)	36(19)	136 ( 51)							188 ( 81)
	***68.8***	***52.8***	***37.5***							***43.1***

2 Axis	160 ( 71)	8 ( 1)	141 ( 25)	5 ( 4)	6 (3)					351 (104)
	***44.4***	***12.5***	***17.7***	***80.0***	***50.0***					***29.6***

3 R wave	903 (293)	26 ( 8)	598 (142)	2 ( 1)						1518 (444)
	***32.4***	***30.8***	***23.7***	***50.0***						***29.2***

4 ST depression	61 ( 49)	120 (70)	72 ( 37)	278 (87)						528 (243)
	***80.3***	***58.3***	***51.4***	***31.3***						***46.0***

5 T abnormality	25 ( 22)	240 (116)	226 (103)	99(38)	276 (78)					849 (357)
	***88.0***	***48.3***	***45.6***	***38.4***	***28.3***					***42.0***

6 A-V conduction	3 ( 2)	3 ( 2)	187 (63)	12 ( 2)	34 ( 7)					237 ( 76)
	***66.7***	***66.7***	***33.7***	***16.7***	***20.6***					***32.1***

7 Ventricular conduction	20 ( 13)	120 (63)	188 (66)	5 ( 2)	228 (46)	0				558 (190)
	***65.0***	***52.5***	***35.1***	***40.0***	***20.2***	***0***				***34.1***

8 Arrhythmia	120 ( 33)	2 ( 2)	62 (43)	2 ( 0)	0	7 ( 5)	133 (28)	162 (43)	496(148)	984 (302)
	***27.5***	***100.0***	***69.4***	***0***	***0***	***71.4***	***21.1***	***26.5***	***29.8***	***30.7***

9 Miscellaneous	97 ( 41)	343 (74)	9 ( 5)	4647 (927)	505 (117)					5601(1164)
	***42.3***	***21.6***	***55.6***	***19.9***	***23.2***					***20.8***

8-9-1 Premature beats <10%: 239(104) ***43.5 %*** See text for code 5-5

9-4-1 Counter clockwise rotation: 3954(709) ***17.9 %***, 9-4-2 Clockwise rotation: 693(218) ***31.2 %***

The control group without major ECG codes: 5,535 (836) ***15.1%***

### Hazard ratios of 5 risk factors among subjects along with any one of major ECG codes (Table 2)

With analysis using Cox proportional hazard models, the death hazard ratios of subjects with any one of major ECG findings were formulated in Table 2, adjusted for sex, age, systolic blood pressure, blood glucose, and smoking habits. The hazard ratio of females to males was 0.65. By separate analysis by sex, the hazard ratio of subjects with abnormal ECG among males was 1.34, and tended to be higher than those (1.19) in females. The hazard ratios tended to be higher in females than those in males for smoking habits (1.29 vs 1.18).

**Table 2.** Hazard ratios of major risk factors to all cause mortality among the subjects in the 19 years of follow-up period, 1980 to 1999, NIPPON DATA 80.

Risk factors included	Both sexes	Males	Females
Sex (M/F)	0.65 (0.52-0.74) ***	-	-
Age (year)	1.12 (1.11-1.12) ***	1.11 (1.11-1.12) ***	1.12 (1.12-1.13) ***
Systolic blood pressure (mmHg)	1.004 (1.002-1.006) ***	1.004 (1.001-1.007) **	1.004 (1.001-1.007) *
Blood glucose (mg/dl)	1.003 (1.002-1.004) ***	1.003 (1.002-1.004) ***	1.003 (1.002-1.005) ***
Smoking habits (0-3)^+^	1.22 (1.14-1.318) ***	1.18 (1.10-1.28) ***	1.29 (1.08-1.54) **
Abnormal ECG (0,1) ^++^	1.28 (1.17-1.408) ***	1.34 (1.18-1.52) ***	1.19 (1.04-1.36) *

***: p<0.001 **: p<0.01 *: p<0.05

+ See text This was a multivariate analysis by Cox prooportional hazard model, done by sexs.

++ Number (deaths) in Abnormal ECG group with any major ECG code: 4,103 (1,174): 28.6% died.

95 % confidence intervals in parenthesis.

### Effect by exclusion of the deaths close to the date of examination (Table 3)

The Cox hazard ratios of mortality from all causes were essentially similar for the subjects with abnormal ECG finding before (1.28) and after excluding the deaths within 5 years after the examination (1.21). Those of sex, systolic blood pressure, blood glucose, or smoking did not change appreciably.

Based on these results, the further analysis by Cox proportional hazard model was performed with all deaths throughout the follow-up period.[Table tbl03]

**Table 3. tbl03:** Hazard ratio of major risk factors to all cause mortality before and after exclusion of the deaths within 3 and 5 years just after the examination in 1980, NIPPON DATA 80.

	1980 - 1999	1983 - 1999	1985 - 1999
Sex (M/F)	0.645 (0.582-0.714) ***	0.648 (0.583-0.721) ***	0.631 (0.564-0.706) ***
Age (year)	1.117 (1.112-1.122) ***	1.117 (1.112-1.122) ***	1.117 (1.111-1.122) ***
Systolic blood pressure (mmHg)	1.004 (1.002-1.006) ***	1.004 (1.002-1.007) ***	1.004 (1.002-1.007) ***
Blood glucose (mg/dl)	1.003 (1.002-1.004) ***	1.003 (1.002-1.004) ***	1.003 (1.002-1.004) ***
Smoking habits (0-3)^+^	1.219 (1.136-1.308) ***	1.216 (1.129-1.309) ***	1.217 (1.127-1.315) **
Abnormal ECG (0,1)^++^	1.276 (1.165-1.398) ***	1.245 (1.113-1.368) ***	1.210 (1.096-1.335) *

***: p<0.001 **: p<0.01 *: p<0.05

+: See text

++: Abnormal ECG: Any major electrocardiographic finding by Minnesota Code: 0=no, 1=yes.

### Hazard ratio of subjects with any one of Q-QS, axis deviation, and high R codes (Table 4)

**1) Q-QS codes:** The hazard ratios of subjects with each Minnesota Code adjusted for age, systolic blood pressure, blood glucose, smoking habits, and sex if applicable, were shown in Table 4. The ratios for Q-QS code 1-1 were significantly high in both sexes. The ratio for minor Q-QS code 1-3 also showed a significant elevation.

**Table 4.** Hazard ratios of the subjects with major electrocardiographic findings for all cause mortality in 1980 to 1999, NIPPON DATA80.

Minnesota code	Both sexes		Males		Females	
Q-QS 1-1	3.71 (1.78-7.71)	***	3.71 (1.78-7.71)	***	4.46 (1.41-14.05)	*
Q-QS 1-2	1.75 (1.10-2.78)	*	2.22 (1.27-3.86)	**	1.24 (0.51-2.99)	
Q-QS 1-3	1.57 (1.18-2.09)	**	1.59 (1.08-2.34)	*	1.54 (1.01-2.35)	*

Axis 2-1	1.37 (1.07-1.76)	*	1.19 (0.86-1.66)		1.81 (1.57-2.65)	**
Axis 2-2	0.69 (0.10-4.92)		1.22 (0.17-8.68)		-	
Axis 2-3	1.80 (1.21-2.69)	**	1.83 (1.12-2.98)	*	1.71 (0.84-3.47)	
Axis 2-4	2.85 (1.06-7.67)	*	3.36 (1.24-9.09)	*	-	
Axis 2-5	4.16 (1.325-13.05)	*	4.56 (1.45-14.4)	**	-	

High R 3-1	1.34 (1.16-1.54)	***	1.33 (1.08-1.56)	**	1.40 (1.11-1.75)	**
High R 3-2	1.91 (0.95-3.84)		3.39 (1.08-10.6)	*	1.45 (0.60-3.50)	
High R 3-3	1.35 (1.12-1.62)	**	1.30 (1.03-1.55)	*	1.45 (1.08-1.94)	*
High R 3-4	3.56 (0.50-25.38)		6.31 (0.87-45.68)	^+^	-	

ST 4-1	2.59 (1.91-3.52)	***	2.79 (1.85-4.22)	***	2.46 (1.56-3.86)	***
ST 4-2	2.00 (1.55-2.57)	***	2.38 (1.56-3.61)	***	1.91 (1.39-2.64)	***
ST 4-3	1.63 (1.16-2.29)	**	1.55 (0.90-2.65)		1.72 (1.10-2.67)	*
ST 4-4	1.15 (0.92-1.44)		1.35 (0.97-1.87)	^+^	1.00 (0.73-1.37)	

T 5-1	2.33 (1.51-3.61)	***	2.27 (1.24-4.16)	**	2.53 (1.34-4.78)	**
T 5-2	1.82 (1.49-2.22)	***	2.52 (1.86-3.42)	***	1.43 (1.09-1.87)	**
T 5-3	1.54 (1.24-1.91)	***	1.62 (1.14-2.31)	**	1.56 (1.18-2.05)	**
T 5-4	1.35 (0.96-1.88)	^+^	1.45 (0.90-2.32)		1.26 (0.78-2.03)	
T 5-5	1.06 (0.84-1.34)		1.02 (0.68-1.52)		1.05 (0.79-1.41)	

AV 6-1	2.01 (0.50-8.10)		4.16 (0.58-29.93)		1.22 (0.17-8.70)	
AV 6-2	7.82 (1.95-31.39)	**	14.29 (1.97-104.0)	**	5.37 (0.75-38.25)	^+^
AV 6-3	1.23 (0.95-1.60)		1.38 (1.02-1.87)	*	0.98 (0.58-1.64)	
AV 6-4	1.16 (0.29-4.65)		1.33 (0.33-5.36)		-	
AV 6-5	2.21 (1.05-4.66)	*	3.71 (1.38-9.97)	**	1.41 (0.45-4.41)	
					1.47 (0.61-3.57)	
V 7-1	2.11 (1.22-3.67)	**	3.06 (1.50-6.24)	**	1.85 (1.19-2.89)	**
V 7-2	1.44 (1.11-1.88)	**	1.21 (0.87-1.68)		1.02 (0.70-1.50)	
V 7-3	1.20 (0.93-1.55)		1.37 (0.97-1.92)	^+^	-	
V 7-4	2.20 (0.55-8.87)		3.23 (0.80-13.13)		0.80 (0.50-1.27)	
V 7-5	1.03 (0.76-1.38)		1.16 (0.78-1.71)			
					1.44 (0.92-2.23)	
AR 8-1	1.92 (1.45-2.54)	***	2.41 (1.67-2.49)	***	-	
AR 8-2	2.14 (0.53-8.64)		2.41 (0.59-9.80)		2.76 (1.85-4.11)	***
AR 8-3	2.42 (1.77-3.31)	***	1.94 (1.16-3.22)	*	1.36 (0.50-3.67)	
AR 8-6	1.31 (0.54-3.16)		0.92 (0.13-6.57)		0.99 (0.60-1.64)	
AR 8-7	1.34 (0.97-1.97)		2.73 (1.51-4.93)	***	0.63 (0.26-1.53)	
AR 8-8	1.29 (0.94-1.75)		1.48 (1.06-2.07)	*		
					0.91 (0.64-1.28)	
AR 8-9-1	1.45 (1.18-1.79)	***	2.07 (1.58-2.72)	***		
					1.28 (0.87-1.87)	
M 9-1	1.47 (1.07-2.03)	*	1.70 (0.93-3.11)	^+^	0.98 (0.37-2.64)	
M 9-2	1.33 (1.04-1.71)	*	1.32 (1.02-1.71)	*	-	
M 9-3-1	1.76 (0.73-4.27)		1.89 (0.78-4.59)		2.07 (0.86-5.01)	
M 9-3-2	1.45 (0.90-2.36)		1.36 (0.77-2.42)		0.97 (0.82-1.14)	
M 9-4-1	1.08 (0.96-1.22)		1.18 (1.01-1.39)	*	1.36 (1.07-1.72)	*
M 9-4-2	1.47 (1.26-1.71)	***	1.55 (1.26-1.90)	***	1.11 (0.62-1.99)	
M 9-5	1.28 (1.05-1.56)	*	1.29 81.04-1.60)	*		

***: p < 0.001 **: p < 0.01 *: p < 0.05 +: p < 0.1.

AV: Atrioventricular V: Ventricular AR: Arrhythmia; M: Miscellaneous codes

Adjusted for age, systolic pressure, blood glucose, smoking habits, and sex if applicable.

95 % confidence interval in parenthesis.

**2) Axis deviation codes:** The hazard ratio for axis code 2-1 (left axis deviation) was 1.37 for male and female combined. The ratio was not significant in males (1.19), contrary to that in females (1.81).

The ratios of subjects with axis code 2-3 (minor right axis deviation) were significantly high among males and females combined and among males. The hazard ratio for axis code 2-4 (extreme axis deviation, 2.85) and 2-5 (4.16) was significantly high among the sex combined and among males, and tended to be higher than those codes for 2-1, 2-2, or 2-3.

**3) High R codes:** The hazard ratios for codes 3-1 and 3-3 (left high R codes) were significantly high at the same levels. The hazard ratio for code 3-2 was significantly high only in males. The ratio for code 3-2 tended to be higher than those for code 3-1 and code 3-3 in males.

### Hazard ratio of subjects with any one of ST depression and T abnormality codes (Table 4)

**1) ST depression codes:** Among the hazard ratios for code 4-1, 4-2, 4-3, and 4-4 (ST depression), those for code 4-1 and code 4-2 were significantly high in the sex combined, in males, and in females. The hazard ratios of subjects with ST depression were ordered inversely by the second code numbers in the sex combined, in males and in females.

**2) T abnormality codes:** Among the hazard ratios of subjects with T abnormality, that for code 5-1 was the highest in females and in the sex combined. The ratio of code 5-2 was the highest in males. The hazard ratios for code 5-4 and for code 5-5 were not statistically significant.

### Hazard ratio of subjects with any one of atrio-ventricular and ventricular conduction defect codes (Table 4)

**1) Atrio-ventricular codes:** Among the death hazard ratios of subjects with incomplete atrio-ventricular block, that of code 6-2 was the highest in males and in females. The hazard ratio for code 6-5 (short PQ interval) was also significantly high in males or females. The ratio for code 6-3 (PQ prolongation) was significantly high only in males.

**2) Ventricular codes:** Among the death hazard ratios of subjects with intraventricular block code (code 7-1, 7-2, 7-3, 7-4, and 7-5), those for code 7-1 and 7-2 were significantly high. Although the ratio for code 7-4 was also high but not significant in males and in the sexes combined. The hazard ratio of subjects with complete right bundle branch block (code 7-2) was significant only in females and in the sexes combined.

### Hazard ratio of subjects with any one of arrhythmia and miscellaneous codes (Table 4)

**1) Ectopic beats:** The hazard ratio of subjects with frequent ectopic beats (code 8-1) was over double but not significant in males and close to double in the sex combined. The hazard ratio with less frequent ectopic beats (code 8-9-1, an optional code for the National Survey) was also over double and significant in males.

**2) Atrial fibrillation or flutter:** Among the hazard ratios of subjects with arrhythmia, code 8-3 (atrial fibrillation or flutter) was the highest in females and in the sexes combined. The hazard ratios for code 8-7(sinus tachycardia) and code 8-8 were significantly high only in males, but not in females at all.

**3) Miscellaneous codes:** The death hazard ratio of subjects with low voltage (code 9-1) was significantly high only in the sex combined. The hazard ratio of subjects with ST elevation (code 9-2) was significantly high in the sexes combined and in males, but not in females. The hazard ratio of subjects with counter clockwise rotation (code 9-4-1) was significantly high only in males, and that with clockwise rotation (code 9-4-2) was significantly high in each sex and in both sexes combined. The ratio with high T wave (code 9-5) was significantly high in males and in the sexes combined.

### Deaths from heart disease among the subjects with ECG codes (Table 5)

The proportion of heart disease death in the abnormal ECG group in females tended to be higher than that in males (21.9 vs 17.2 %). The proportions of death from heart diseases tended to be higher in males with Q-QS abnormality, atrio-ventricular conduction defect, and ventricular codes than in females. Although the number of cases was limited, it was true of the deaths from ischemic heart disease.

However, the percentage proportions of death from heart diseases tended to be higher in females with the other ECG code groups, especially left high R, ST depression, T abnormality, ectopic beats, atrial fibrillation or flutter, low voltage, or even counter clock or clock-wise rotation codes. The death proportion of deaths from cerebral infarction tended to be high in females with atrial fibrillation or flutter, but not so high in males (25.9% vs 12.5 %).

## DISCUSSION

One of major contributions of this study comes from the extensive national samples residing in exactly 300 stratified randomly sampled areas which were sub-samples defined by the Statistical Information Bureau, Ministry of Health and Welfare (Ministry of Health, Labour, and Welfare after reorganization in 2001) for several national surveys in 1962, 1972, 1980, 1990, and 2000. The over-all response rate was over 79% in the relevant National Survey on Circulatory Disorders in 1980,^1^ but the response rates in municipal area were lower than those in rural areas.

The high hazard ratio of the subjects with abnormal Q-QS findings showing the possible past history of myocardial infarction was observed even considering for sex, age, systolic blood pressure, blood glucose, and smoking habits. In the subjects with Q-S finding, proportion of death from heart disease was two third of all deaths and that was almost double to those in the reference subjects (Table 5). This result corresponds to the fact that a subject with history of myocardial infarction has the higher risk of recurrence.

**Table 5.** Death proportions among subjects with selected ECG codes by selected disease categories, 1980-1999, NIPPON DATA80.

	No. of deaths	Proportion (%)					

		Cerebrovascular diseasae	Cerebral infarction*	Cardiovascular disease	Ischemic heart disedase**	Cancer	Others
	Males						
Control group	405	18	10	14	7	35	33
Abnormal ECG group	686	17	10	17	6	31	35

Q-QS abnormality (mc1-1 to 1-3)	50	12	6	34	12	20	34
Axis deviation (mc2-1 to 2-5)	41	17	15	17	5	22	44
Left High R (mc3-1,3-3)	287	20	13	15	7	30	35
ST depression (mc4-1 to 4-4)	115	19	12	22	8	24	36
T abnormality (mc5-1 to 5-5)	150	17	11	25	7	23	35
Atrioventricular codes (mc6-1 to 6-5)	56	14	7	23	13	38	25
Ventricular codes (mc7-1 to 7-5)	116	17	8	19	8	31	33
Ectopic beats (mc8-1,8-9-1)	99	15	8	21	6	29	34
Atrial fibrillation (mc8-3)	16	13	13	19	0	31	38
Low voltage (mc9-1)	11	18	9	9	0	27	46
ST elevation (mc9-2)	70	14	9	21	9	30	34
Counter clock rotation (mc9-4-1)	348	16	10	12	5	37	36
Clock rotation (mc9-4-2)	132	17	8	22	7	32	30
High T (mc9-5)	107	17	11	10	5	38	35

	Females						
Control group	431	16	8	17	7	31	37
Abnormal ECG group	488	19	11	22	8	21	38

Q-QS abnormality (mc1-1 to 1-3)	31	19	3	29	13	13	39
Axis deviation (mc2-1 to 2-5)	30	27	10	17	3	20	37
Left High R (mc3-1,3-3)	148	23	12	26	10	16	35
ST depression (mc4-1 to 4-4)	128	23	13	30	14	17	31
T abnormality (mc5-1 to 5-5)	207	20	11	28	12	17	35
Atrioventricular codes (mc6-1 to 6-5)	20	25	5	10	0	20	45
Ventricular codes (mc7-1 to 7-5)	74	19	11	11	4	22	49
Ectopic beats (mc8-1,8-9-1)	58	17	12	33	12	17	33
Atrial fibrillation (mc8-3)	27	33	26	33	4	11	22
Low voltage (mc9-1)	30	10	0	23	3	13	53
ST elevation (mc9-2)	4	0	0	25	25	25	50
Counter clock rotation (mc9-4-1)	361	15	7	21	9	26	38
Clock rotation (mc9-4-2)	86	17	9	30	9	16	36
High T (mc9-5)	10	20	20	30	10	50	0

Minnesota codes (mc) in parencesis

* : Secondary mention of cerebrovascular disease.

**: Secondary mention of cardiovascular disease.

Although prevalence of the extreme axis deviation (code 2-4, n=5) or indeterminate axis (code 2-5, n=6) were relatively rare, the observed high hazard ratios of early death might come from any disposition of the heart or abnormal propagation of the excitation due to any serious heart disease. The genesis of indeterminate axis was suggested as a posterior, rightward and superior orientation of terminal QRS forces, which might result from number of causes, by a quantitative vectorcardiographic analysis by Goldberger.^21^

The high Cox hazard ratios of subjects with left high R (code 3-1 or code 3-3), due to hypertension, cardiomyopathy, valvular disease, or sports heart as well as merely thin chest, show the significance of myocardial hypertrophy for prevention of early deaths. The Cox hazard ratio of subjects with right high R (code 3-2) was the higher than those of code 3-1 and code 3-3, though significance was lower maybe due to fewer cases with 3-2 code. The highest but not significant hazard ratio was observed for subjects with bi-ventricular high R (code 3-4) which was added in the revised Minnesota Codes 1982.^3^

The hazard ratio of subjects with ST depression was higher than that of those with T abnormality. Because ST depression code should be with T abnormality by definition in Minnesota Codes,^2^ the hazard ratio of subjects with ST depression may show a combination effect of ST and T abnormalities. Besides coronary atherosclerosis, ST depression with T abnormality might be developed in hypertension. ST depression might be assumed as a sign of arterio-sclerosis and/or hypertensive stress leading to the relevant complication such as not only heart disease but also cerebrovascular disease, finally to death, as shown in Table 5.

One of the striking facts was that the T abnormality was always more frequent in females than males in almost all of Japanese communities. However, the hazard ratios of subjects with T abnormality (code 5-1 to code 5-3) were high in males and in females. The hazard ratio of subjects with T abnormality code 5-4 was high but not significant in this analysis. The code 5-5, minimally low T, was added according to Japanese scientists to Minnesota codes.^18^ However, this study could not support the significance at the moment.

The hazard ratio of subjects with complete left bundle branch block was higher than that of complete right bundle branch block in males. However, the ratio of complete right bundle branch block in female was significantly high and larger than that of complete left bundle branch block.

It was reasonable that the hazard ratio of subjects with frequent ectopic beats was higher than that of less frequent ectopic beats (code 8-9-1). The frequent ectopic beats might lead to atrial fibrillation in case of supraventricular beats, and ventricular fibrillation in case of ventricular beats. The hazard ratio of atrial fibrillation was comparable with major high-risk codes such as Q-QS, ST depression, and T abnormality.

Resting heart rate was an independent predictor of 16.5-year death in this same Japanese cohort reported by Okamura et al.^10^ The significant relation was observed only in males in this study, as shown in Table 4. The association of heart rate with coronary heart disease was reported in males and females, particularly striking in black women according Gillum et al. without any specific explanation.^14^

The Cox hazard ratio of subjects with ST elevation code 9-2 was significantly high in males and in the sex combined, but not in females as shown in Table 4. Recently Brugada syndrome, which included a type of ST elevation, has been presented as a new insight,^22^ and so further analysis of subjects with code 9-2 should be done for their prognoses in detail.

It is noteworthy that the hazard ratio of subjects with clockwise rotation (code 9-4-2) was significantly high and close to that of low voltage, atrial conduction defects, or high T wave. The observed significantly high hazard ratio for code 9-4-2 was a surprise because we did not expect any prognostic significance in this code beforehand. The mechanism or the reason should be clarified by a further analysis as far as possible.

According to the definition of Cox proportional hazard model,^20^ the calculated hazard ratios would go up or down by those confounding factors, such as sex, age, systolic blood pressure, blood glucose, smoking habits, and the other factors. Naturally the hazard ratio to early deaths should be evaluated by considering all of those risk factors included in this analysis. The one practical way to apply these results was to evaluate over-all death risk done directly by a computer and one of the other ways would be with using multidimensional table including typical levels of these risk factors, which will appear in a later paper.

Naturally the sex difference of the results comes from the biological difference as well as their social life difference, such as social stress, eating habits, education.

The effect of the removal of deaths within 5 year after the examination on the hazard ratios was examined. Though the other confounding factors, such as age, systolic blood pressure, blood glucose, and smoking habits, showed a trivial or no significant effect, any of major ECG findings might contribute more in some cases of deaths in the early phase of observed period.

The cause of deaths among the subjects with any ECG code might be interesting and was demonstrated as the percentage proportion in Table 5. The highest death hazard ratios of heart diseases were observed in the subjects with Q-QS findings, and then with T abnormality and also with ST depression. It was impressive that the hazard ratios of subjects with ST elevation were comparable with those of ST depression code 4-4, considering recent reports of Burgada syndrome.^22^ It was worth to note that the death proportions from the other cause of deaths among the subjects with low voltage were relatively higher, though the hazard ratios were significant only in the sexes combined. We are going to analyze and to discuss the relationship in details and will publish the results before long.

This study demonstrated a series of striking results using a national sample to give clear data the relationship of ECG findings objectively diagnosed to deaths from all causes. These data would be the base to prevent early deaths by intervening as a national project, such as the Health Japan 21 or Healthy people 2010 in the United State. Although we could not discuss much about the subjects group of small size, there would be a starting point for further studies.

The morbidity hazard ratio of specific disease, such as cerebral stroke and myocardial infarction, would be much more interesting along with mortality, but it was very hard to get such morbidity data of the subjects throughout country at the moment.

In this analysis, each specific ECG finding was evaluated using the Cox proportional hazard model for deaths of all cause, and any combination of ECG findings would show a much higher significant hazard ratio. Some of the further analysis on combination of ECG findings will come in our later papers.

## Appendix I: NIPPON DATA80 Research Group. (In alphabetical order by the family name of principal investigators, but not by the associates)

NIPPON DATA80: Abbreviation of “National Integrate Projects for Prospective Observation of Non-communicable Diseases And its Trend in the Aged, based on the data of the National Survey in 1980”

1. ***Chairperson:*** Hirotsugu Ueshima (Department of Health Science, Shiga University of Medical Science, Otsu, Shiga)

2. ***Consultant:*** Osamu Iimura (Hokkaido JR Sapporo Hospital, Sapporo, Hokkaido)

3. ***Principal investigators:*** Tsutomu Hashimoto (Department of Public Health, Wakayama Medical College, Wakayama), Hiroshi Horibe (Aichi Medical University, Aichi), Minoru Iida (Osaka Medical Center for Cancer and Cardiovascular Diseases, Osaka), Kazunori Kodama (Department of Epidemiology, Radiation Effects Research Foundation, Hiroshima), Masumi Minowa (Department of Epidemiology, National Institute of Public Health, Saitama), Akira Okayama (Department of Preventive Cardiology, National Cardiovascular Center, Osaka), Koryo Sawai (Japanese Association for Cerebro-cardiovascular Disease Control, Tokyo), Shigeo Shibata (Clinical Nutrition, Kagawa Nutrition University, Saitama), Shigemichi Tanaka (Department of Cardiology, Cardiovascular Center, Teine Keijinkai, Hokkaido), Kazuo Ueda (Division of Internal Medicine, Kyushu University School of Health Science, Fukuoka), and Hiroshi Yanagawa (Saitama Prefecture University, Saitama).

4. ***Associate members:*** Katsuhiko Kawaminami (Department of Epidemiology, National Institute of Public Health, Saitama), Toshihiro Takeuchi, Mitsuru Hasebe, Fumitsugu Kusano with staff members in 300 public health centers, Japan, Fumiyoshi Kasagi (Department of Epidemiology, Radiation Effects Research Foundation, Hiroshima), Yoshikuni Kita, Takehiko Hayakawa, Sohel R. Choudhury (Department of Health Science, Shiga University of Medical Science, Shiga).

## Appendix II: (List of principal collaborators and their associates in the Working Group for ECG Coding in the National Survey on Circulatory Disorders, 1980 in alphabetical order by the family name of principal collaborators with their institution in 1980)

1. ***Moderator:*** Hiroshi Horibe (National Cardiovascular Center, Osaka)

2. ***Principal collaborators:*** Mitunori Doi (Tosa-Yamada Public Health Center, Kochi), Syuichi Hatano (National Institute of Public Health, Tokyo), Masamitsu Konishi (National Cardiovascular Center, Osaka), Yasushi Morisawa (Dokkyo University School of Medicine, Tochigi), Chiaki Sasade (Takikawa Public Health Center, Hokkaido), Koryo Sawai (Japanese Association for Cerebro-Cardiovascular Disease Control, Tokyo), Shigeo Shibata (Kagawa Nutrition University, Saitama), Takashi Shimamoto (University of Tsukuba School of Medicine, Ibaraki), Hironori Toshima (Kurume University School of Medicine, Fukuoka), Hiroshi Yanagawa (Jichi Medical University, Tochigi), Tsutomu Hashimoto (Jichi Medical School, Tochigi)

3. ***Associate collaborators:*** Takashi Kato (Aichi Medical University, Aichi), Takahiro Usami (Dokkyo University School of Medicine, Tochigi), Kazuaki Shimamoto (Sapporo Medical University, Hokkaido), Kazuo Suzuki (Akita Institute of Cerebrovascular Disease, Akita), Yoshihiko Watanabe (Fujita Health University School of Medicine, Aichi), Takashi Watanabe (National Ohta Hospital, Shimane)
